# Human Growth Factor/Immunoglobulin Complexes for Treatment of Myocardial Ischemia-Reperfusion Injury

**DOI:** 10.3389/fbioe.2022.749787

**Published:** 2022-02-28

**Authors:** Benjamin Liebman, Claire Schwaegler, Andrea T. Foote, Krithika S. Rao, Taylor Marquis, Alexander Aronshtam, Stephen P. Bell, Prospero Gogo, Richard R. LaChapelle, Jeffrey L. Spees

**Affiliations:** ^1^ Department of Medicine, Cardiovascular Research Institute, University of Vermont, Colchester, VT, United States; ^2^ Pharmacology Graduate Program, University of Vermont, Burlington, VT, United States; ^3^ Cellular and Molecular Biomedical Sciences Program, University of Vermont, Burlington, VT, United States

**Keywords:** growth factor, protein complex, HGF, FGF2, IgG, reperfusion injury, ischemia

## Abstract

Hepatocyte Growth Factor (HGF) and Fibroblast Growth Factor 2 (FGF2) are receptor tyrosine kinase agonists that promote cell survival after tissue injury and angiogenesis, cell proliferation and migration during tissue repair and regeneration. Both ligands have potential as systemic treatments for ischemia-reperfusion injury, however clinical use of HGF and FGF2 has been limited by poor pharmacokinetic profiles, i.e., their susceptibility to serum proteases, rapid clearance and short half-lives. Previously, we reported vaso- and cardioprotective protein complexes formed between HGF and polyclonal, non-specific immunoglobulin (IgG) with therapeutic efficacy in a rat model of myocardial ischemia with reperfusion (MI/R). Here, using a pre-clinical porcine MI/R model, we demonstrate human HGF/IgG complexes provide significant myocardial salvage, reduce infarct size, and are detectable in myocardial tissue 24 h after intracoronary injection. Furthermore, we show that multiple daily infusions of HGF/IgG complexes after MI do not lead to production of HGF-specific auto-antibodies, an important concern for administered biologic drugs. In experiments to identify other growth factors that non-covalently interact with IgG, we found that human FGF2 associates with IgG. Similar to human HGF/IgG complexes, FGF2/IgG complexes protected primary human cardiac endothelial cells under simulated ischemia (1% oxygen and nutrient deprivation) for 48–72 h. Molecular modeling studies suggested that FGF2 and HGF both interact with the Fc domain of IgG. Also, we tested whether an Fc-fusion protein would bind FGF2 to form complexes. By native gel electrophoretic assays and biochemical pulldowns, we found that Jagged1, a Notch1 ligand that controls stem cell self-renewal and tissue regeneration, bound FGF2 when presented as a Jagged1- Fc fusion protein. Our results suggest that human growth factor/IgG and FGF2/Fc- fusion complexes have potential to provide a biologics platform to treat myocardial ischemia-reperfusion and other forms of tissue injury.

## Introduction

Cardiovascular disease is the leading cause of death worldwide, accounting for 31% of all deaths and the loss of nine million people annually (Virani et al., 2020). Of these deaths, about 50% are attributed to coronary heart disease (Smith et al., 2015). Coronary heart disease (CHD) is the umbrella term for pathologies affecting the ability of the epicardial coronary arteries to provide oxygenated blood and nutrients to the heart (Vogel et al., 2019). Significant and sustained imbalance between coronary oxygen supply and demand results in myocardial ischemia and the progressive death of cardiomyocytes, termed myocardial infarction or MI (Eisen, Giugliano et al., 2016; Anderson and Morrow, 2017). In the United States, the estimated annual incidence of MI is 605,000 new heart attacks and 200,000 recurrent heart attacks; this equates to an American suffering a heart attack every 40 s (Virani et al., 2020).

Rapid and early reperfusion of ischemic myocardium prevents cardiomyocyte death and has been the most effective treatment for preserving jeopardized myocardial tissue and limiting infarct size (Virani et al., 2020). Accordingly, the current standard of care is coronary revascularization, achieved through primary Percutaneous Coronary Intervention (PCI) (O’Gara et al., 2013) or administration of fibrinolytic agents (e.g., Alteplase, Tenecteplase), which enzymatically break down blood clots (Schneider and Sobel, 2008). PCI typically involves catheterization of the femoral or radial artery and catheter insertion into the occluded coronary artery, followed by inflation of an angioplasty balloon with an expandable wire cage (stent), which mechanically opens the vessel. The stent remains in place to stabilize the damaged artery after the balloon is deflated and the catheter is removed. Primary PCI is the most common strategy for revascularization and preferred over fibrinolytic therapy for patients when time-to- treatment delays are relatively short (<120 min), treatment at hospital with a catheterization laboratory is available, and the duration of ischemia is <12 h (O’Gara et al., 2013; Bagai et al., 2014).

Revascularization of the epicardial coronaries is important for patient survival after MI. Paradoxically, however, myocardial reperfusion also causes injury, killing vascular endothelial cells, cardiomyocytes and other vulnerable cells, which greatly reduces the benefits of reperfusion therapy (Yellon and Hausenloy, 2007). Lethal reperfusion injury, or the injury and eventual death of cardiomyocytes that were viable immediately prior to reperfusion, contributes up to 50% of final infarct size (Garcia-Dorado et al., 2009). In addition to PCI and thrombolysis to treat MI, reperfusion injury also occurs during coronary artery bypass (CABG) procedures and heart transplantation (Verma et al., 2002).

Growth factor-based treatments for ischemia-reperfusion and tissue injury have been studied in numerous animal models and proof-of-concept clinical trials that demonstrated positive safety profiles. But most growth factors administered intra-arterially or IV exhibited unfavorable pharmacokinetics (e.g., short half-lives and rapid clearance) and limited efficacy (Appasamy et al., 1993; Lazarous et al., 1997; Simons et al., 2002; Yang et al., 2009). Treatment with factors such as fibroblast growth factor 2 (FGF2) or hepatocyte growth factor (HGF) is highly attractive due to their potential for synergistic signaling through common protective and pro-survival pathways such as Ras/MAPK and/or PI3K/Akt (Liao et al., 2007; Banquet et al., 2011; Koraishy et al., 2014; Barć et al., 2020). The engagement of these pathways in myocardium with infarction has potential to protect vulnerable cardiomyocytes and endothelial cells from death, reduce vascular leak, and encourage angiogenesis for repair and regeneration.

Previously, our research group identified HGF/immunoglobulin (IgG) complexes in medium that was conditioned by human epicardial-derived cells (Rao et al., 2015). HGF/IgG protein complexes that we formed by ultracentrifugal concentration of recombinant, active human HGF and polyclonal IgG uniquely protected vascular endothelial cells from ischemic injury by signaling through c-Met, the HGF receptor, and by triggering phosphorylation of “related to tyrosine kinase” (RYK), an orphan Wnt co-receptor. Furthermore, in a rat model of myocardial ischemia with reperfusion, we found that intra-arterial treatment with HGF/IgG complexes significantly improved vascular integrity and cardiac function (Rao et al., 2015).

Here we provide additional *in vivo* data for myocardial salvage, infarct size, and distribution and retention of HGF/IgG complexes using a pre-clinical, large animal model with PCI, as well as data showing lack of immunogenicity following multiple administrations after MI with reperfusion. Also, we report the development of two new growth factor protein complexes comprised of low molecular weight (18 kDa) human FGF2 with IgG or Jagged1-Fc fusion protein.

## Results

### Localization of HGF/IgG Complexes in Myocardial Tissue 24 h Following Infusion From PCI Guide Catheter After MI/R

**FIGURE 1:**
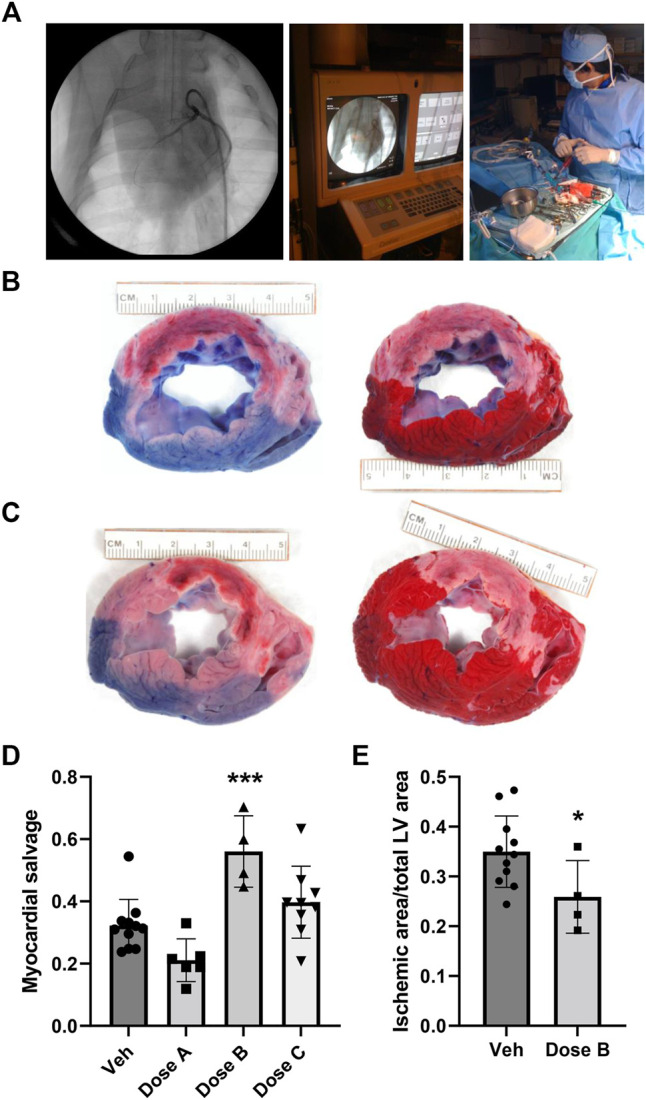
Intracoronary administration of human HGF/IgG complexes from the PCI guide catheter in a pre-clinical porcine model of MI with reperfusion. **(A)** Left, middle: Representative images from fluoroscopic angiography showing guide catheter with contrast dye. Right: Intracoronary infusion of HGF/IgG complexes or vehicle from the indwelling guide catheter at time of reperfusion (60 min post-ischemia). Pigs were treated with 62.5 µg HGF and 105 µg mixed polyclonal IgG, representing a 1:1: molar ratio. **(B,C)** Left: At 24 h after MI, reperfusion, and treatment, Evan’s blue dye stain shows areas with adequate perfusion (blue) and low perfusion (pink; a.k.a. area at risk, AAR) in representative vehicle-treated heart section **(B)** and HGF/IgG-treated heart section **(C)**. Right: TTC stain shows living muscle tissue (red) and necrotic, dying tissue (white; a.k.a. ischemic area, IA). **(D)** Quantification of myocardial salvage following MI/R and treatment with vehicle or three different doses of HGF/IgG complexes (Dose A, 31.25 µg HGF/52.5 µg IgG; Dose B, 62.5 µg HGF/105 µg IgG; Dose C, 125 µg HGF/210 µg IgG. One-way ANOVA with multiple comparisons. ***p < 0.001. Myocardial salvage = AAR-IA/AAR. **(E)** Reduction in area of myocardial tissue with infarction, 24 h after MI/R and treatment with Dose B. Data are mean ± SD. n = 4–11 pigs per group. 2-tailed, unpaired T test. *p = 0.05.

When administered IV, HGF has a plasma half-life of ∼3.8 min and is primarily taken up by the liver (Appasamy et al., 1993). To determine whether intracoronary delivery of human HGF/IgG complexes would improve retention and/or tissue distribution, as opposed to rapid clearance, we performed MI/R surgery in pigs. To mimic a clinical delivery scenario, we used a femoral artery access and PCI guide catheters, wires, angioplasty balloons and stents commonly used for interventional procedures at UVM Medical Center. Under fluoroscopic guidance, we advanced the catheters through the aorta and into the left anterior descending (LAD) (Figure 1A). By injecting contrast dye, we estimated the relative degree of dominance for the ventricular blood supply coming from the left circumflex artery and the LAD. Accordingly, the balloon was placed over the guide wire at the first or second diagonal branch of the LAD, inflated, and then left inflated for 60 min to occlude the artery and produce ischemic injury. Immediately upon reperfusion, we infused 12.5 ml of DMEM medium (vehicle) or 12.5 ml DMEM with HGF/IgG complexes into the coronary from the indwelling guide catheter. The total time of infusion was ∼2 min.

After 24 h, pigs were intubated and re-anesthetized. Prior to euthanization, a suture was used to ligate the LAD at the proximal end of the stent and Evan’s blue dye was infused to delineate areas of perfusion and risk (Figures 1B,C). Hearts were then removed, sliced at 1 cm thickness, and stained with TTC to identify areas of necrosis (Figures 1B,C). The sections were digitally-photographed and fixed in formalin for paraffin processing, sectioning and histology. Using Scion Image software, an observer blinded to sample/slide ID determined the area at risk (AAR) and ischemic area (IA), as well as total left ventricle (LV) area. We assayed hearts from pigs treated with three different doses of HGF/IgG, each matched at a 1:1 molar ratio. Compared with vehicle, a single intracoronary dose containing 62.5 μg of human HGF with 105 μg porcine IgG provided a significant degree of myocardial salvage (AAR-IA/AAR) at 24 h after MI/R and intracoronary treatment (Figure 1D). The 62.5 μg HGF dose also significantly reduced infarct size, as shown by ischemic area (i.e., necrotic tissue)/total LV area (Figure 1E).

**FIGURE 2:**
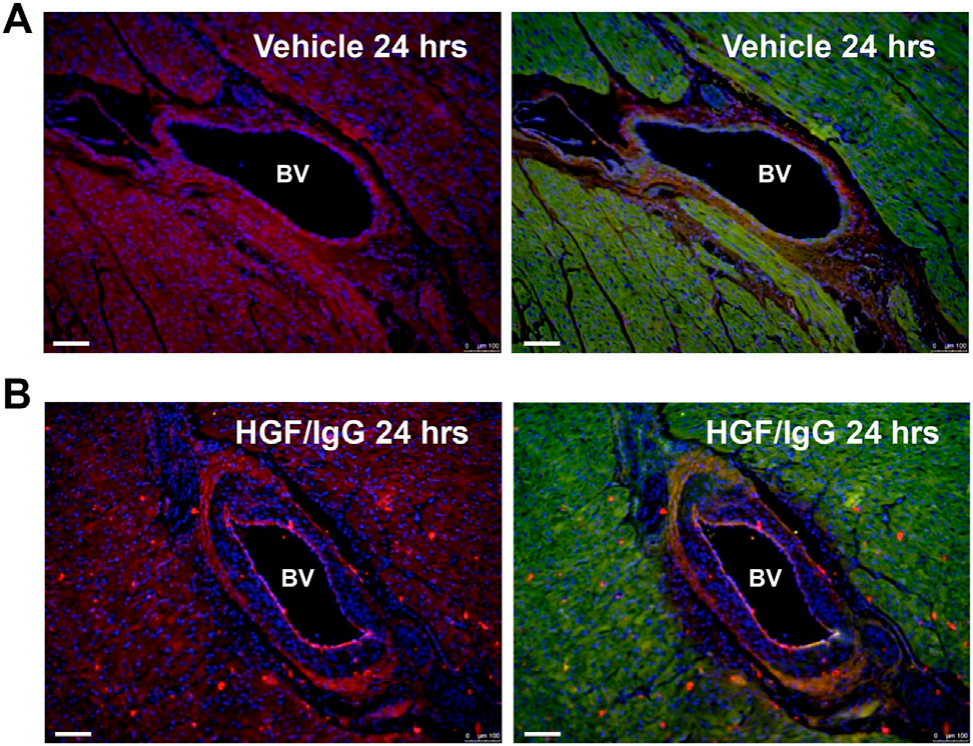
Vascular and myocardial distribution of HGF/IgG complexes 24 h after intracoronary infusion. Note red dots (ALEXA 594-conjugated secondary antibody) on endothelial wall and throughout myocardium in **(B)** that are not present in vehicle-treated heart **(A)**. Prior to staining with secondary antisera and DAPI (blue, nuclei), both **(A)** and **(B)** were stained with polyclonal primary antisera against 6x His-tag to detect recombinant human HGF. Hearts in **(A)** and **(B)** are representative of n = 3 pigs per group that were stained.

We used immunohistochemistry with antisera against the 6x-His tag on our recombinant human HGF to detect the HGF/IgG complexes in tissue sections (Figures 2A,B). Staining of left ventricular myocardial tissue bordering zones with infarction demonstrated a wide-spread distribution of HGF/IgG complexes; this pattern was associated with the microvasculature and arterioles as well as within myocardial tissue (n = 3 pigs, Figure 2B). As expected, similar staining of vehicle-treated control pigs did not identify any complexes and lacked the spotty pattern of complexes throughout the myocardium (n = 3 pigs, Figure 2A).

### Multiple IV Infusions of HGF/IgG Complexes After MI Do Not Induce Production of Circulating HGF-Specific Auto-Antibodies

Autoimmune reaction is an important concern for clinical application of antibody-based biologics (Her and Kavanaugh, 2016). To examine the potential immunogenicity of HGF/IgG complexes in the context of MI with reperfusion, we performed a set of experiments in Sprague Dawley rats to test for the production of auto-antibodies directed against HGF. Groups of adult rats underwent surgery to temporarily occlude the LAD artery for 2 h, followed by reperfusion. With the use of echocardiography, we confirmed cardiac injury in all rats 1 week after MI, and M-mode images obtained at 2, 3, and 4 weeks after MI demonstrated decreased LV wall thickness, consistent with myocardial damage and necrosis (data not shown).

**FIGURE 3:**
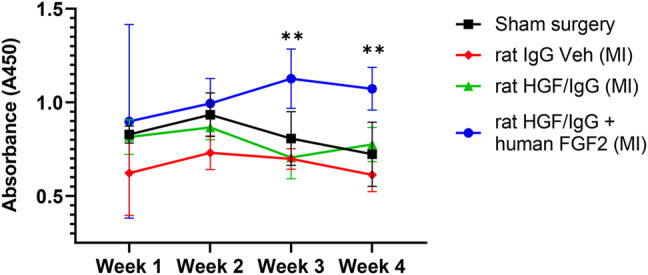
Multiple administrations of HGF/IgG complexes do not induce HGF- specific auto-antibodies in rats subjected to myocardial ischemia-reperfusion. After MI/R surgery or sham surgery, blood plasma was obtained from rats weekly and assessed for circulating antibodies specific to rat HGF. Animals received three separate treatments: one at reperfusion, one on day 3, and one on day 5 post-MI/R with either vehicle (containing IgG), or HGF/IgG. HGF/IgG-treated animals showed no auto-immune reactivity to HGF. However, weekly addition of human FGF2 to HGF/IgG complexes (positive xenogenic control) induced antibodies to rat HGF, as shown by a mild immune response at weeks 3 and 4. Data represent mean ± SD and were analyzed by repeated measures two-way ANOVA. n = 5 animals, **p < 0.01.

To form HGF/IgG complexes, we mixed recombinant rat HGF with polyclonal rat IgG and concentrated 40-fold by centrifugation with an ultracentrifugal filter unit. As a positive control for immune reactivity, one group of rats received rat HGF/rat IgG with recombinant human FGF2 added to the complexes. We expected that multiple administrations with a xenogenic protein mixture would facilitate detection of circulating antibodies directed against rat HGF (i.e., auto-antibodies). Immediately after reperfusion, we administered HGF/IgG complexes, HGF/IgG complexes with human FGF2, or IgG alone (vehicle control) to three groups of rats. All treatments were infused directly into the LV lumen through the apex (i.e., intra-arterial). Subsequently, all rats received two additional “booster” injections by tail vein on days 3 and 5 after MI. Blood plasma was obtained at weekly intervals post-surgery for 4 weeks. Using a modified ELISA system, we determined that animals treated with rat HGF/IgG (n = 5) exhibited no autoimmune activity at any of the time points analyzed (Figure 3). By contrast, compared with vehicle-treated controls, rats infused with rat HGF/IgG complexes and human FGF2 (n = 5) exhibited a mild immune response at 3 and 4 weeks following MI and treatment (Figure 3). These results indicate administered growth factor/IgG complexes may be well-tolerated, particularly with a limited number of infusions and if species-matched for immune compatibility.

### Identification of FGF2/IgG Complexes That Protect Human Cardiac Microvascular Endothelial Cells During Simulated Ischemia

Previously, we demonstrated that a mixture of HGF and IgG (1:1 molar ratio) formed protein complexes after 40-fold concentration by centrifugation in Amicon ultracentrifugal filter units (Rao et al., 2015). To investigate whether FGF2 interacted non-covalently (i.e., electrostatically) with IgG, we similarly incubated human FGF2 (LMW isoform, 18 kDa) with IgG in a 1:1 molar ratio and incubated the mixture for 30 min at room temperature. Following 40-fold concentration with Amicon units, the samples were evaluated by native agarose gel electrophoresis as in Rao et al. (2015).

**FIGURE 4:**
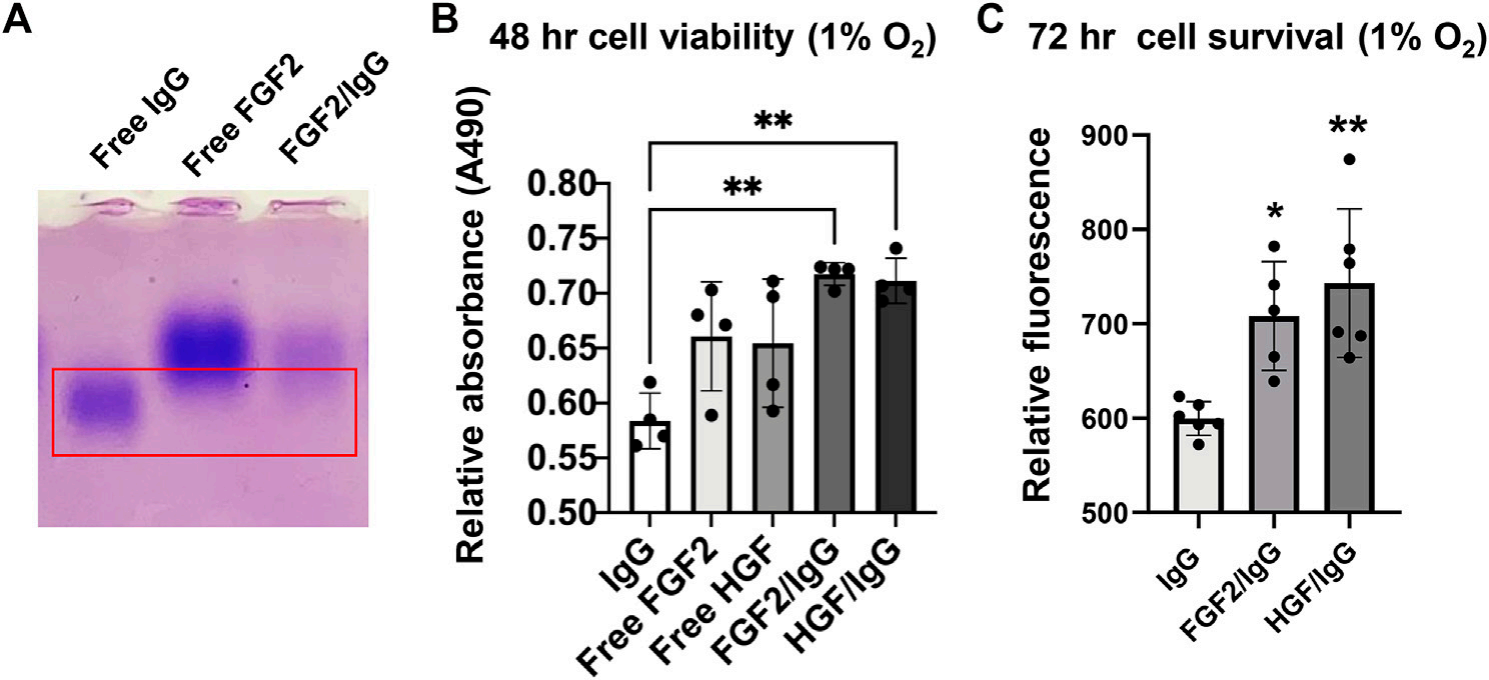
FGF2/IgG complexes protect primary human cardiac microvascular endothelial cells against simulated ischemia for 48–72 h. **(A)** Native isoelectric focusing gel to detect protein complexes. Lanes: 1) free IgG (alone); 2) free FGF2 (alone); 3) FGF2/IgG complexes. Gel was stained for 30 min with Coomassie brilliant blue and de-stained overnight in 10% acetic acid. Red box: note that the protein band expected for free IgG is absent in the right-hand lane with FGF2/IgG complexes. **(B)** MTS assay demonstrated increased cell viability conferred by FGF2/IgG and HGF/IgG complexes relative to IgG alone (matched dose) after 48 h of exposure to 1% oxygen and nutrient deprivation. Data are mean ± SD, n = 4 experimental replicates. *p < 0.05, compared with IgG. **(C)** CyQuant assays showed that FGF2/IgG and HGF/IgG complexes protected human cardiac microvascular endothelial cells against simulated ischemia for 72 h. Data are mean ± SD and were analyzed by one-way ANOVA with *post-hoc* comparisons. n = 6 experimental replicates. **p < 0.01, compared with IgG.

During native gel electrophoresis, protein separation occurs primarily by differences in isoelectric point rather than by size. After electrophoresis of free FGF2, free IgG, and FGF2/IgG protein samples in 1.3% agarose gels (50 mM MES buffer; pH, 7.0–8.5), staining with Coomassie Brilliant Blue dye demonstrated that the higher isoelectric point of FGF2 (pI = 9.58) resulted in a slower migrating species compared with the migration pattern observed for free IgG (pI = 6.6–7.2) (Figure 4A). Of special interest, on gel lanes with concentrated FGF2/IgG mixtures, we saw a distinct band shift, whereby FGF2 reduced the migration distance for free IgG in the gel (Figure 4A). A series of different gel runs demonstrated that the interaction of FGF2 with IgG occurred across a range of pH (7.0–8.5) (data not shown). Furthermore, by solid phase binding assays, we observed a dose-responsive increase in low molecular weight FGF2 binding to IgG (Table 1).

**TABLE 1:** Dose-responsive increase in human FGF2 binding to immobilized IgG. Numbers represent mean absorbance (450 nm) and SD values from human FGF2 ELISA. Assays were run in duplicate.

Input	FGF2	
IgG	4 ng/ml	10 ng/ml
100 ng/ml	0.014 ± 0.011	0.042 ± 0.001
500 ng/ml	0.04 ± 0.003	0.071 ± 0.006
10,000 ng/ml	0.193 ± 0.148	0.46 ± 0.045

Signaling by FGF2 and HGF increases the survival of many different primary and transformed cell types (Nakamura and Mizuno, 2010; Ornitz and Itoh, 2015). In light of increased endothelial cell survival and vascular integrity conferred by HGF/IgG after MI/R (Rao et al., 2015), we tested FGF2/IgG in cell protection assays with primary human cardiac microvascular endothelial cells under culture conditions that simulated ischemia (nutrient deprivation and 1% oxygen). By MTS assay, a measure of cell metabolism, FGF2/IgG complexes significantly improved endothelial cell viability during 48 h of exposure compared with cells that were incubated in a matched concentration of IgG (Figure 4B). In agreement, quantification of cell survival using a nucleic acid dye-binding assay (Cyquant Direct) after 72 h of simulated ischemia demonstrated a significant increase in survival of microvascular endothelial cells incubated in FGF2/IgG complexes (Figure 4C). Together, these assays indicated that FGF2/IgG complexes provided a similar level of microvascular endothelial cell protection as did HGF/IgG complexes, under matched experimental conditions (Figures 4B,C).

### PatchDock/PyMOL Modeling of Human Growth Factor/IgG Interactions

**FIGURE 5:**
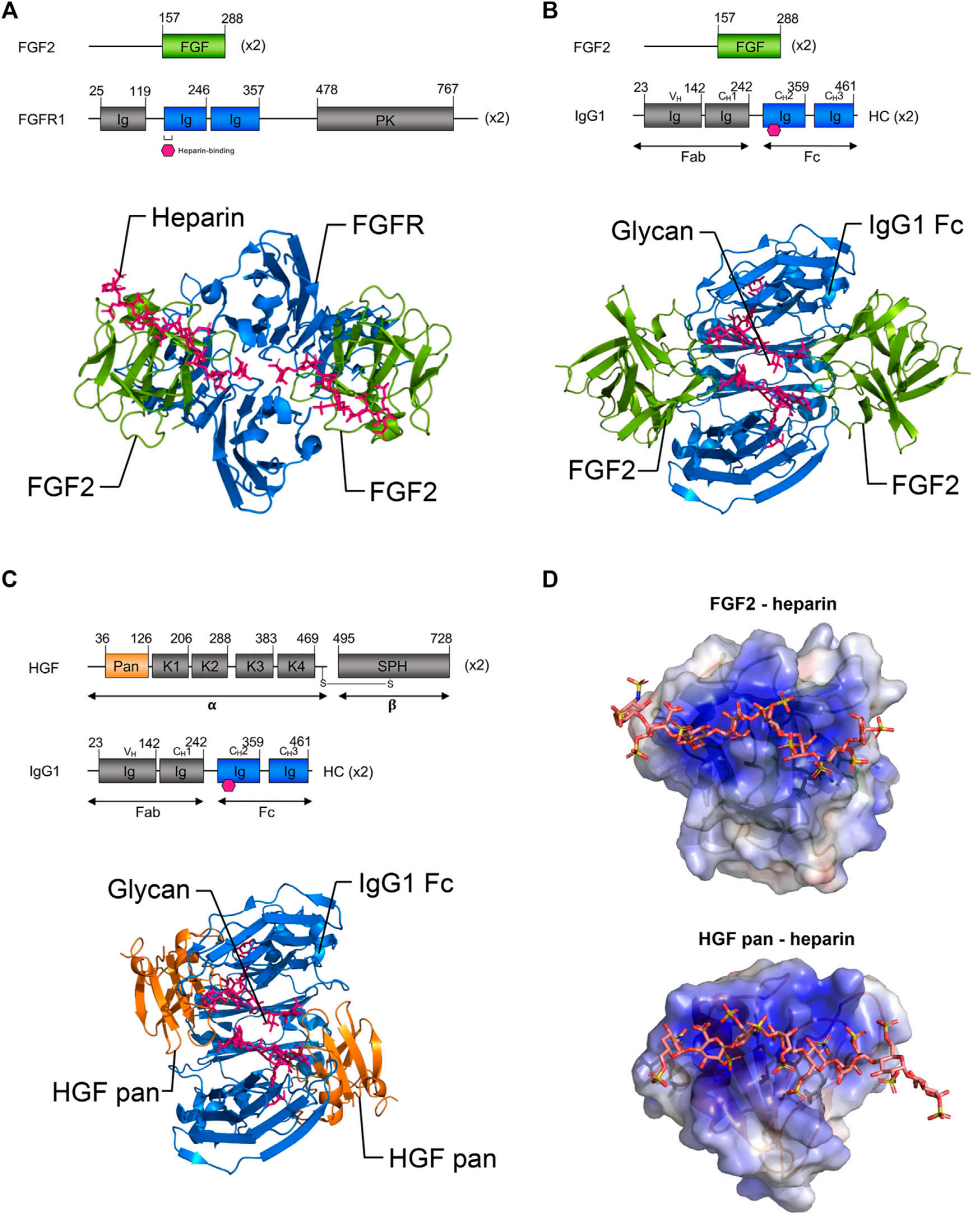
Three-dimensional modeling of proposed growth factor: IgG complexes. **(A)** Top: Schematic representations of full-length FGF2 and FGFR1. Bottom: Ribbon representations of FGF2 (residues 158–286, green) bound to FGFR1 (blue) and heparin (pink) (published crystal structure, PDB ID 1FQ9). Note the binding of FGF2 proteins to FGFR1 occurs within immunoglobulin-like sub-domains. **(B)** Top: Schematic representations of the proposed binding of FGF2 to the IgG1 Fc domain. Bottom: Ribbon model of proposed binding of FGF2 (green) to the Fc domain of IgG1 (blue) within FGF2/IgG complexes. Note that based on similarities to FGF2:FGFR1 binding, it is possible that the Fc domain of IgG may accommodate the binding of two FGF2 ligands. **(C)** Top: Schematic representations of the HGF pan domain and IgG1. Bottom: Ribbon model of proposed binding of HGF pan domain (orange) to the Fc domain of IgG1 (blue) in HGF/IgG complexes. Note that it is also possible that the Fc domain of IgG may accommodate the binding of two HGF ligands. **(B,C)** Pink hexagon (top) and sticks (bottom) = glycosylation. **(D)** Electrostatic surface potential models of FGF2 and HGF pan domain bound to heparin (sticks) (PDB IDs 1FQ9 and 1GMO, respectively). Note growth factors have heparin binding domains that are thought to bind within immunoglobulin-like domains of their respective RTK receptors. Basic patches (blue surface) may be involved in the binding of FGF2 and HGF in the described complexes. PK, protein kinase; HC, heavy chain; K1–K4, Kringle domains; SPH, serine protease homology domain; alpha, HGF alpha chain; beta, HGF beta chain. Gray, domains not shown in ribbon diagrams.

Binding of FGF2 ligands to their cognate receptor(s) FGFR1, 2, 3 is facilitated by heparan sulphate proteoglycan co-factors (Ornitz and Itoh, 2015). Binding occurs within IgG-like subdomains of the receptor, followed by receptor dimerization; this enables the receptor to bind two FGF2 ligands (Schlessinger et al., 2000). Given the dimeric binding of FGF2 to FGFR, we were curious if the FGF2/IgG complexes mimicked endogenous ligand-receptor binding and whether polyclonal IgG could structurally accommodate two FGF2 molecules. To ascertain the probable conformations of FGF2 and HGF in complex with IgG, we generated 3-dimensional protein docking models based on available crystal structures. We compared our FGF2/IgG model to a published crystal structure of FGF2 with heparin glycan co-factor bound to FGFR1 (FGFR1 PDB ID:1FQ9; Figure 5A).

In docking experiments to model the geometry of FGF2/IgG complexes (Figure 5B), the IgG1 crystal structure (PDB ID: 1HZH) was prepared in PyMOL, by removing waters and Fab fragments, leaving only the Fc dimer (residues 244–478 of 1HZH). This PDB file was uploaded to the PatchDock server as the “receptor” molecule (Schneidman-Duhovny et al., 2005). Next a single molecule of FGF2 (chain A from PDB ID 1CVS, residues 158–286 of the full-length 288 amino acid isoform 1) was uploaded as the “ligand” molecule. Clustering RMSD and complex type were both set to default before submitting the run. The top 100 solutions, scored based on surface geometric complementarity and area of contact, were retrieved. The first six high-scoring poses were eliminated as they placed the N- and/or C-termini of FGF2 facing into the surface of IgG. In this configuration, the full length FGF2 protein would extend from these residues and clash with IgG. Therefore, the seventh highest ranked pose was selected for use in our model (Figure 5B).

A docking experiment to model possible interactions between HGF pan domain and IgG Fc was performed in a manner similar to that of the first docking experiment (Figure 5C). First, IgG1 Fc (PDB ID: 1HZH with waters and Fab fragments removed) was uploaded as the “receptor” molecule. Then, the HGF pan domain (chain A from PDB ID: 3HMS, corresponding to residues 36–126 of the full-length 728 amino acid HGF isoform 1, was then uploaded to PatchDock as the “ligand” molecule, and the docking parameters were set as before. The top three poses were eliminated as in the first docking experiment due to clashes that would exist between full-length HGF and IgG. The fourth highest ranking pose was therefore selected for our model (Figure 5C).

The majority of docking solutions placed FGF2 or HGF pan at the joint between the first and second Ig domains of the Fc region, with nearly an equal number on either of side of the Fc region of IgG in between the CH3 and CH2 domains. Notably, FGF2 and HGF both have heparin binding domains that are thought to bind within immunoglobulin-like domains of their respective RTK receptors (Spivak-Kroizman et al., 1994). Both FGF2 and HGF pan domain are roughly spherical and are similar in size, suggesting their associations with IgG could be spatially comparable.

The glycosylation sites on the CH3 Ig domain within the Fc portion of IgG are similar in position and size to the heparin-binding sites within the second IgG-like domain of FGFR. Thus, it is plausible that IgG glycosylation may function in a manner analogous with heparin. In agreement with this concept, electrostatic surface potential models generated using the APBS plugin for PyMOL of the growth factor/IgG complexes highlighted the potential for interactions at heparin binding domains (Figure 5D).

### Complexes Formed Between Human FGF2 and Jagged1-Fc

**FIGURE 6:**
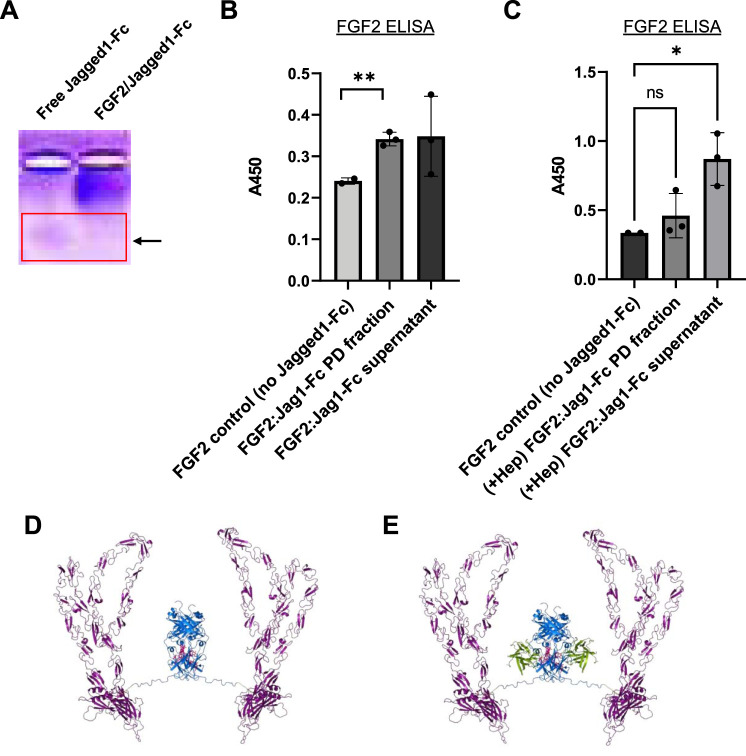
Generation of recombinant human FGF2/Jagged1-Fc complexes. **(A)** Native isoelectric focusing gel to detect protein complexes. Lanes: 1) free Jagged1- Fc fusion protein (alone); 2) FGF2/Jagged1-Fc complex. Red box: note that the protein band expected for free Jagged1-Fc IgG is absent in the right-hand lane with FGF2/Jagged1-Fc complexes. Thus, complexation with FGF2 retards the migration of Jagged1-Fc. **(B)** ELISA data for FGF2 to assay complex formation. FGF2 was incubated either alone or with Jagged1-Fc-His, followed by addition of anti-His-biotin. Streptavidin-agarose was used to pulldown complexes and 0.5% Sodium deoxycholate was used to dissociate protein-protein interactions prior to FGF2 ELISA. Data in **(B,C)** are mean ± SD and were analyzed by one-way ANOVA with *post-hoc* comparisons. n = two to three experimental replicates. **p < 0.01. **(C)** Addition of excess heparin (50 μg) during initial incubation of FGF2 with Jagged-Fc-His prevents complex formation. **p < 0.01. **(D)** Ribbon representation of modeled Jagged1-Fc fusion protein homodimer. **(E)** Ribbon representation of modeled Jagged1-Fc fusion in complex with FGF2.

We next asked whether fusion of the Fc domain from IgG1 to proteins of interest could be utilized to form new protein complexes. Although IgG1 Fc is currently used in many FDA-approved biologics to improve drug half-life and reduce clearance, in this case, we hypothesized here that the Fc domain would allow Jagged1, a Notch1 receptor ligand, to interact in a novel way with FGF2. In concept, growth factors or cytokines that do not naturally bind IgG could potentially expressed as Fc-fusion proteins, and then combined with HGF or FGF2 to form complexes. In support of this hypothetical system, native gel electrophoresis of concentrated mixtures of FGF2/Jagged1-Fc demonstrated a shift in migration pattern relative to Jagged1-Fc alone (Figure 6A).

To confirm complex formation with FGF2 and Jagged1-Fc, we performed a series of biochemical pulldown experiments. Taking advantage of the 6x histidine purification tag on Jagged1-Fc, we combined FGF2 and Jagged1-Fc at an estimated equimolar ratio. Following a 2 h incubation at room temperature and storage overnight at 4°C, we incubated the samples for 2 h with anti-his-biotin. Then, streptavidin-agarose beads and centrifugation were used to pulldown the Jagged1-Fc. Following several washes, 0.5% Sodium Deoxycholate was used to dissociate the putative complexes and FGF2 ELISA was used to detect FGF2 that had complexed with Jagged1-Fc. By FGF2 ELISA, we detected significantly more FGF2 in pulldowns where FGF2 was incubated with Jagged1-Fc relative to control pulldowns, which contained FGF2 but lacked Jagged1-Fc (Figure 6B). In support of the hypothesis that the Fc domain of IgG1 interacts with FGF2 in a manner similar to heparin, addition of excess heparin during incubation of FGF2 and Jagged1-Fc prevented complex formation; this resulted in FGF2 remaining in the supernatant rather than being pulled down by streptavidin-agarose (Figure 6C).

To visualize a mode of Jagged1-Fc interaction with FGF2 consistent with our FGF2/IgG Fc docking results, we built a model of Jagged1-Fc fusion protein (catalog # 1277-JG, R&D Systems, Minneapolis, MN, United States) using the AlphaFold v2.0 human Jagged-1 model (Ser32-Ser1046) spliced to human IgG1 (residues corresponding to Pro100-Lys330 of Uniprot accession # P01857 of the 1HZH structure), via an Ile-Glu-Gly-Arg-Met-Asp linker using PyMOL (Senior et al., 2020) (Figures 6D,E). Our native gel and pulldown results, and models, illustrate how an Fc fusion protein and FGF2 can be combined to form unique protein complexes. Notably, this strategy has exciting potential to provide a customizable platform to create new biologic drugs.

## Discussion

Our results in adult pigs demonstrated that intra-arterially administered HGF/IgG complexes distributed throughout the myocardium after PCI and stenting and were retained for at least 24 h after MI/R. Of three intracoronary HGF doses tested, we found that the middle dose with 62.5 μg human HGF and 105 μg porcine IgG was most effective at providing myocardial salvage and reducing infarct size. Furthermore, multiple infusions of species-matched HGF/IgG complexes after MI/R did not induce an immune response against HGF. Thus, as a formulation, HGF/IgG complexes may enhance retention of HGF and the duration of cytoprotective receptor signaling.

HGF is composed of α-subunit and β-subunits that are joined by a disulfide linkage. Functional domains within the subunits include an N-terminal hairpin domain, four kringle domains, and a catalytically inactive serine protease domain (Niemann, 2013). The canonical HGF receptor c-MET is a heterodimeric protein composed of a 50 kD extracellular α-chain and a 145 kD β-chain. In addition to the extracellular region, the β-chain also contains a single-pass transmembrane domain and a cytoplasmic tail which contains the active tyrosine kinase domain (Organ and Tsao, 2011). Analysis of crystal structures of HGF/c-MET showed HGF binding occurs within the β-chain, which contains a heparin sulphate binding domain (Organ and Tsao, 2011). HGF induces dimerization of c-MET, followed by tyrosine kinase activity and autophosphorylation, which triggers the recruitment of downstream adaptor molecules. Signaling through the HGF/c-MET axis results in signal propagation through multiple downstream adaptor pathways including: PI3K/Akt, Ras/Raf/MAPK, JAK/STAT and Wnt/β-Catenin (Organ and Tsao, 2011; Holland et al., 2013; Koraishy et al., 2014; Zhang et al., 2018). HGF signaling regulates tissue growth and morphogenesis during development, as well as cell migration, survival, angiogenesis and fibrosis during repair/remodeling after injury (Bottaro et al., 1991).

In a rat model of MI, Ono et al. reported elevated plasma levels of HGF were present 60 min following reperfusion (Ono et al., 1997). HGF and c-MET mRNA levels increased in the heart 3-fold at 24–48 h following reperfusion, and remained elevated for 120 h. Nakamura et al. reported treatment with recombinant HGF was cardioprotective against ischemia/reperfusion injury in rats (Nakamura et al., 2000), and reduced cardiomyocyte death and infarct size compared with controls. In contrast, administration of neutralizing antisera against endogenous HGF resulted in increased cardiac cell death and infarct expansion (Nakamura et al., 2000).

FGF2 has multiple isoforms ranging from 18 to 34 kDa that enable a wide array of possible quaternary structures that affect protein function (Yu et al., 2007). Several high molecular weight isoforms of FGF2 promote fibrosis after tissue injury. By contrast, low molecular weight FGF2 (18 kDa) and HGF are both reported to reduce fibrosis (Wang et al., 2004; Liao et al., 2007; Koo et al., 2018). Fibroblast Growth Factor Receptors (FGFRs) are tyrosine kinases activated by the binding of FGF ligands and heparan sulphate proteoglycans (HSPGs), which act as co-factors (Yayon et al., 1991). The presence of HSPGs potentiates FGFR activity and also enhances ligand binding via a high affinity binding site (Ornitz and Itoh, 2015). HSPGs exist as cell surface-bound proteoglycans, transmembrane proteoglycans, or, are bound by the extracellular matrix (Matsuo and Kimura-Yoshida, 2013). FGF2-FGFR binding results in signal transduction through multiple pathways including RAS-MAPK, PI3K-AKT, PLCγ and STAT (Eliceiri, 2001; Liao et al., 2007; Ahmad et al., 2012). FGF2 signaling plays fundamental roles in skeletal and neural development and controls the proliferation, differentiation, and survival of numerous adult somatic cell types. Also, it regulates critical processes such as angiogenesis and fibrosis during tissue remodeling/repair (Karajannis et al., 2006; Kardami et al., 2007; Müller et al., 2012).

In a canine model of MI, intracoronary injection of FGF2 was shown to reduce infarct size and improve cardiac function (Yanagisawa-Miwa et al., 1992). Furthermore, the authors reported that FGF2 treatment promoted myocardial angiogenesis, and resulted in increased vessel density in the FGF2-treated group compared with vehicle-treated control animals (Yanagisawa-Miwa et al., 1992). In isolated mouse hearts subjected to 60 min ischemia/reperfusion, specific knockout of low molecular weight FGF2 reduced capacity for recovery and cardiac performance relative to that of wild type mice (Liao et al., 2007).

Intra-arterially administered FGF2 has been tested in clinical trials for patients with atherosclerotic peripheral arterial disease and intermittent claudication (Lazarous et al., 1997) as well as cardiac atherosclerosis (Simons et al., 2002). Although one or two treatments with FGF2 was well-tolerated, efficacy was limited. For example, in the phase II FGF Initiating RevaScularization Trial (FIRST), a double-blind, randomized, and controlled study, a single intracoronary infusion of FGF2 was given to patients with severe coronary artery disease (Simons et al., 2002). Notably, however, treatment with FGF2 at 0, 0.3, 3, or 30 µg/kg did not improve exercise tolerance, the primary clinical endpoint (Simons et al., 2002). Lack of success in several trials was attributed, in part, to unsatisfactory pharmacokinetic profiles for FGF2 (Bush et al., 2001). Similar to other growth factor proteins, FGF2 is quickly eliminated from the circulation, with a serum half-life of 50 min and poor target tissue distribution (Lazarous et al., 1997). Efforts directed at improving the pharmacokinetic profile of FGF2 may increase its therapeutic potential. For example, treatment with FGF2 encapsulated into sustained-release heparin-alginate coated capsules during coronary bypass graft surgery resulted in improved myocardial perfusion and a near complete cessation of angina related symptoms (Ruel et al., 2002).

In regard to the non-covalent interaction of HGF and FGF2 with polyclonal, non-specific IgG, it is notable that both growth factors bind within IgG-like domains of their respective receptors. Also, ligand-receptor interaction for both growth factors is potentiated by heparin and heparan sulphate proteoglycans embedded within the ECM (Chirgadze et al., 1999; Schlessinger et al., 2000; Basilico et al., 2008). The fusion of the Fc domain of IgG to proteins prolongs serum half-life, reduces clearance, and has been widely adopted for the production of therapeutic biologics. For example, Etanercept, an FDA-approved tumor necrosis factor receptor: Fc fusion protein, is indicated for multiple inflammatory conditions such as rheumatoid arthritis and plaque psoriasis (Scott, 2014). The improved pharmacokinetic profile of Fc fusion proteins is primarily due to Fc domain binding to the neonatal receptor for IgG (FcRn) (Roopenian and Akilesh, 2007; Suzuki et al., 2010; Czajkowsky et al., 2012). Similar to Fc-fusion proteins, the distribution and retention of HGF/IgG and FGF2/IgG complexes in the heart and other target tissues may benefit from interactions with FcRn.

The pro- and anti-inflammatory activities of IgGs are modulated through interaction of the IgG Fc domain with distinct IgG Fc receptors (FcγRs) on cells (Nimmerjahn and Ravetch, 2008). Heavy chain glycosylation of IgG Fc domains determines, in part, whether antibodies can bind FcγRs on immune cells to stimulate antibody-dependent cellular cytotoxicity (Shinkawa et al., 2002). Previous work suggests that IgG Fc core glycosylation, predominantly with sialic acid sugar moieties, may act as an “immunological switch,” imparting anti-inflammatory properties to IgGs (Kaneko et al., 2006; Anthony and Ravetch, 2010). In this regard, it is of great interest to determine the anti- or pro-inflammatory properties of FGF2/IgG and HGF/IgG *in vivo* and whether the complexes impact local immune cell responses in myocardial tissue after MI/R injury. Importantly, in the case that IgG Fc core glycosylation state is integral to formation and biological activity of growth factor/IgG and FGF2/Fc-fusion complexes, there may be opportunity to tune the properties of complexes based on the glycosylation state of the Fc domain.

Extensive progress in the areas of biomaterials and bioengineering has improved growth factor delivery to tissues/organs with the purpose of promoting tissue repair and regeneration. Examples include scaffolds composed with biodegradable hydrogels or other substrates and seeded with paracrine-acting, reparative cells or purified growth factors. Diverse permutations and designs are possible, and hydrogels may be further modified with extracellular matrix components, drugs, microparticles or nanoparticles (Bruggeman et al., 2016; Xu et al., 2018; Bruggeman et al., 2019; Dong et al., 2020; Roy et al., 2021). Whereas we used a systemic, intra-arterial route here to administer growth factor/IgG complexes, it seems plausible that therapeutic complexes could be embedded within bioengineered substrates or matrices to control timing and localization of release for different applications. Further study will be required to determine the complement of receptors and signaling pathway(s) engaged by FGF2/IgG and FGF2/Jagged1-Fc fusion protein complexes and whether or not they are cardio- and vasoprotective or improve vascular integrity or angiogenesis after MI/R. In addition to treating MI, growth factor/IgG complexes or FGF2/Fc-fusion complexes may be well suited to improve tissue survival and function after organ transplantation or to treat other forms of injury such as diabetic neuropathy, peripheral artery disease, chronic ulcer, and stroke.

## Materials and Methods

### Purification of Recombinant Human Growth Factors

To facilitate cell adhesion, 150 mm^2^ plates (Nunc, Denmark) were pre-coated with human fibronectin (5 μg/ml in PBS). For production of recombinant human HGF, a stable HEK293 HGF-producer cell line (Rao et al., 2015) was grown in DMEM/F12 medium supplemented with 5% FBS (Atlanta Biologicals, Lawrenceville, GA, USA). Medium was changed every 2 days until the cells reached confluence, after which the media was switched to serum-free DMEM/F12. We collected cell-conditioned medium (Cdm) after 24 and 48 h. To remove cell debris, Cdm was filtered (0.2 μm) and stored at −80°C. For purification by SP-Sepharose cation exchange chromatography, the Cdm was adjusted to pH 6.5 to facilitate HGF binding to resin. Cdm was loaded onto a SP-sepharose column that was prepared with an equilibration buffer: 20 mM sodium phosphate (pH 6.5) buffer with 0.02% Tween-80. The column was washed with five volumes of equilibration buffer and then five volumes of equilibration buffer with 0.05 M NaCl. The bound HGF was eluted using a two-step elution: 1) Low salt (equilibration buffer with 0.4 M NaCl), and 2) High salt (equilibration buffer with 0.8 M NaCl). Peak fractions were determined by Bradford protein assay and human HGF ELISA according to the manufacturer’s instructions (HGF Duoset Kit, R&D Systems).

Peak fractions eluted off SP-sepharose resin were pooled and diluted 1:1 with equilibration buffer: 20 mM sodium phosphate, 300 mM sodium chloride and 10 mM imidazole, pH 7.4. Fractions were then loaded by gravity onto a column with 2 ml of Ni- NTA resin (ThermoFisher Scientific, Waltham, MA, United States). The column was washed with PBS supplemented with 25 mM imidazole (pH 7.4) and eluted with PBS with 300 mM imidazole.

Recombinant human FGF2 (18 kDa) was purified from a bacterial expression system (*Escherichia coli* strain BL21), as reported previously (Miao et al., 2020). Briefly, bacteria were grown in Luria-Bertani (LB) broth with ampicillin. Cells were lysed by sonication and the cell extract was pelleted (3,600 × *g* for 10 min). The supernatant was loaded onto a 1 ml column with Ni-NTA resin (ThermoFisher Scientific, Waltham, MA, United States). FGF2 was eluted with 400 mM imidazole. Protein concentration was determined by Bradford protein assay and protein composition and purity was assessed by SDS-PAGE. The eluate was desalted and loaded onto a 1 ml heparin-sepharose affinity column (GE Healthcare Bio-Sciences, Pittsburg, PA, USA). Bound FGF2 was eluted from the heparin-sepharose column with PBS supplemented with 1.5 M NaCl.

### Human Endothelial Cell Protection Studies Under Simulated Ischemia

Primary human cardiac microvascular endothelial cells (HCMVEC) were from multiple donors and three vendors (Lonza Bioscience, Basel, Switzerland; PromoCell, Heidelberg, Germany; ScienCell, Carlsbad, CA, USA). HCMVEC cells were grown in endothelial cell-specific media with supplements (EGM-2MV SingleQuots, Lonza Bioscience; ECGS, PromoCell; ECGS, ScienCell) containing 5% FCS and PenStrep, at 37°C with 5% CO_2_. Cells were seeded in tissue culture plates (Nunc), expanded as needed and frozen down. For cell protection studies, HCMVEC were used between passages 2 and 4. Primary HCMVECs were plated into 48-well plates (Nunc) at 1 × 10^4^ or 2 × 104 cells/well. Cells were grown at 37°C with 5% CO_2_ in endothelial cell growth medium with 5% FCS. All cells were plated 48 h prior to the start of the experiment. For simulated ischemia (oxygen and nutrient deprivation), cells were briefly washed with PBS to remove serum and the medium was switched to serum-free DMEM/F12 with or without various treatments. Cells were exposed to hypoxia (1% oxygen) in a dedicated, specialized incubator fed by nitrogen gas. After 48 or 72 h, plates were removed from the incubator, briefly rinsed with PBS, and frozen at −80°C. For MTS assay of cell viability, the CellTiter 96 Aqueous One solution Cell Proliferation Assay Kit (Promega, Madison, WI, United States) was used according to the manufacturer’s instructions. For quantification of cell survival, a DNA binding dye was used (CyQuant assay, Molecular Probes Invitrogen, Waltham, MA, United States). To ensure full cell lysis and dye incorporation, cells were subjected to 3× freeze-thaw cycles in lysis buffer. The lysis mixture was loaded into fluoroblock plates (Nunc) and relative fluorescence was determined by plate reader (Synergy HT, Biotek, Winooski, VT, USA).

### Preparation of Rat HGF/IgG Complexes

Recombinant rat HGF (Sino Biological US Inc., Wayne, PA, USA) was diluted to a working concentration of 2 μg/ml in sterile PBS. Non-specific, polyclonal IgG from rat serum (Sigma-Aldrich, St. Louis, MO, United States) was diluted to 1.2 μg/ml in sterile PBS. For each injection, a dose of 10 µg of HGF and 6 µg rat IgG was prepared. The HGF was mixed with the IgG in a total volume of 15 ml and concentrated 40-fold (from 15 ml to 375 µl over 45 min) using an Amicon ultracentrifugal filter unit (10 kDa cut-off filter; Millipore Sigma, Bedford, MA, USA).

### Myocardial Ischemia-Reperfusion Surgery and Treatment With HGF/IgG Complexes in Rats

All animal work was approved by the University of Vermont College of Medicine’s Office of Animal Care in accordance with the American Association for Accreditation of Laboratory Animal Care and National Institutes of Health guidelines. Sprague Dawley rats (8 weeks of age) were anaesthetized with 4% isoflurane (to effect) and endotracheally-intubated. Rats were ventilated (Harvard Apparatus, Holliston, MA, United States) and body temperature was maintained with a heated pad (Gaymar T Pump; Stryker, Kalmazoo, MI, United States). Through a dermal incision, a blunt dissection was performed, and the intercostal muscles were separated. The heart was exposed, the LAD coronary artery was encircled with 10–0 nylon suture, and the LAD was occluded. The occlusion was confirmed by blanching of the anterior wall of the left ventricle. The incision was closed, and the animals were allowed to recover off the ventilator. After 2 h of ischemia, the rats were reintubated under anesthesia, ventilated and the chest wall was reopened. The hearts were exposed, and the suture was released. Reperfusion was confirmed by observing blood flow through the LAD and color returning to the previously blanched area. Immediately after reperfusion, or each rat, 125 µl of PBS containing either rat IgG or rat HGF/IgG complexes was injected through the left ventricle wall and into the LV lumen. After the injection, the chest wall was closed with two to three layers of suture. The rats with MI were returned to the vivarium for recovery and were kept for additional infusions and collection of blood plasma. Subsequently, the animals received tail vein “booster” injections with HGF/IgG or IgG on days 3 and 5 post-operation. For sham-operated animals, the chest wall was opened to visualize the intact pericardium (twice); this approach corresponded to that for the ischemia/reperfusion surgery. In sham-operated animals, the suture was passed under the LAD, but the artery was not ligated. No other surgical manipulations were carried out with the sham-operated animals.

### Modified ELISA for Auto-Antibodies Produced Against HGF/IgG Complexes

Following ischemia-reperfusion surgery, blood was drawn at 1, 2, 3 and 4 weeks time points, centrifuged, and the resulting plasma was collected and stored at −80°C. For the detection of auto-antibodies against rat HGF in blood plasma, assays were performed as previously described (Spees et al., 2004; Gregory et al., 2006). Briefly, 1 μg/ml recombinant rat HGF (Sino Biologics, Beijing, China) suspended in PBS was adsorbed onto high protein-binding plates (Nunc) overnight at 4°C, with shaking. The following day, the plate was blocked in PBS (pH 7.4) with 1% BSA (ThermoFisher Scientific, Waltham, MA, United States) for 2 h at room temperature. Following blocking and additional washes, plasma samples were diluted (50-fold or 100-fold) in blocking buffer (1% BSA in PBS, pH 7.4, 0.2 µm filtered) and incubated overnight at 4°C, with shaking. The next day, the plasma wells were washed and incubated for 2 h with goat anti-rat horseradish peroxidase-conjugated antibodies (1:200, Invitrogen, Waltham, MA, United States). A reference standard curve was created with a polyclonal rabbit anti-HGF antibody (Sigma-Aldrich, St. Louis, MO, United States) and pure rat HGF that was pre-adsorbed to the wells. The reference samples were washed and incubated 2 h with a goat anti-rabbit horseradish peroxidase-conjugated antibody (1:2,000, Sigma-Aldrich, St. Louis, MO, United States). Following PBS washes, the plate was incubated with Ultra TMB substrate solution (ThermoFisher Scientific, Waltham, MA, United States) for 50 min. Following color development, stop solution (2 M sulfuric acid) was added to neutralize the substrate solution and absorbance was determined at 450 nM (Synergy HT; Biotek, Winooski, VT).

### Preparation of HGF/IgG Complexes for Porcine Injections

HGF/IgG complexes were prepared 12–16 h prior to injection and stored at 4–6 °C. A dose of 31.25, 62.5, or 125 µg human HGF and 52.5, 105, or 210 µg mixed polyclonal pig IgG was used for each pig, representing a 1:1 molar ratio for each dose tested. Sterile growth factors and IgG were mixed in a total volume of 5 ml and added to an Amicon centrifugal concentration unit. The mixture was then centrifuged at 1,100 × *g* for 30 min at room temperature. In some cases, complexes were centrifuged for an additional 5 min, until a final volume of 300 µl was reached. The complexes were then stored at 4°C overnight. On the day of injection, complexes were reconstituted in sterile DMEM/F12 at a total volume of 12.5 ml.

### Myocardial Ischemia-Reperfusion Surgery in Adult Pigs and Treatment With HGF/IgG Complexes

All work with pigs was performed under a UVM Institutional Animal Care and Use Committee-approved protocol and followed USDA guidelines. Commercial swine (Barrows, ∼50 kg) were pre-medicated with Meloxicam (0.2 mg/kg, PO) sedated with an injection of ketamine (15–20 mg/kg, IM) and atropine (0.05–0.5 mg/kg, IM), masked with isoflurane (5%), and intubated. Anesthesia was maintained with isoflurane (2.5%, inhaled). Intravenous catheters were placed in each ear for drug and fluid administration. A baseline transthoracic echocardiography (ECHO) was performed. Under sterile conditions, a cut-down was done to access the right femoral artery and a 6F vascular introducer was inserted. A blood sample was taken (20 ml) and heparin administered (300 μl/kg, IV) to prevent clotting during the catheterization procedure. A bolus of amiodarone (50 mg, IV) was given to reduce heart irritability and a bolus of fentanyl (0.05 mg/kg, IV) followed by a fentanyl infusion (0.05 mg/kg/h, IV) was given to reduce the amount of isoflurane required for anesthesia. Under fluoroscopic guidance, a guide catheter was inserted and advanced into the opening of the LAD coronary artery. This was followed by a guide wire that was advanced into the distal LAD. A balloon catheter (with or without a stent) was advanced over the wire and positioned in the LAD. Stents were used to later identify the previous position of the balloon during inflation. An infusion of amiodarone was started (10 mg/kg, IV; over 30 min) to additionally reduce heart irritability. The balloon was inflated to eliminate blood supply to a region of the heart muscle for 60 min. Pigs were monitored by electrocardiogram and pulse-oximetry and provided with fluid and thermal support. In the event that ventricular tachycardia or cardiac arrest occurred during the occlusion, appropriate measures were taken to resuscitate the pig, including intravenous lidocaine, amiodarone, epinephrine, and electrical defibrillation.

The balloon was deflated, and reperfusion was confirmed by injection of a bolus of contrast dye through the guide catheter. DMEM/F12 (vehicle control) or protein complexes in vehicle were infused into the LAD from the indwelling guide catheter. Treatments were administered by manual injection in the LAD artery [a.k.a. intracoronary (12.5 ml)]. Following occlusion, physiological parameters and cardiac arrest events were carefully recorded for all pigs. When present, ventricular fibrillation events were promptly addressed with DC cardioversion. The catheter was removed and the femoral artery blood flow restored. After the access site was repaired, additional pain medication was administered (Meloxicam; 0.02 mg/kg, SC), and the animals were monitored continuously until anesthesia wore off and then were transferred to a housing facility.

At 24 h after MI/R surgery and treatment, animals were re-anesthetized and a median sternatomy was performed to gain access to the heart. Umbilical tape snares (1/8 in wide) were placed around the descending aorta, left subclavian artery, and bracheocephalic artery, but not occluded. A silk suture was placed around the LAD artery at the location of the previous balloon inflation, but not occluded. A 7F catheter was inserted into the right carotid artery and the tip advanced beyond the snare into the ascending aorta above the aortic valve. After ventricular fibrillation was induced by brief contact of the myocardium with a 9 V direct current battery, the LAD and snares were occluded. The isolated coronary circulation was perfused with 1.5% Evans blue via the carotid catheter to identify the area at risk. The heart was then removed, sliced at 1 cm thickness with a sharpened brain knife, and photographed to identify the area at risk. Slices were then incubated with 1.5% triphenyltetrazolium (TTC, 30°C) for 25 min and photographed again to identify areas with infarction (white, necrotic).

### Immunohistochemistry

Following Evans Blue/TTC staining of pig hearts, tissue slices were fixed in 10% formalin and paraffin embedding. Ten-micron serial sections were made from blocks of tissue bordering the zone with infarction. Prior to immunohistochemical staining, paraffin was removed by xylenes and alcohol (100%–70%). Tissue sections were washed with PBS and antibody retrieval was performed using 20 μg/ml proteinase K (20 min at RT). Slides were blocked for 1 h in PBS containing 5% normal goat serum and 0.1% Triton X-100, washed with PBS, and incubated at 4°C overnight with primary antibody (anti-6x histidine, 1:1,000, Catalog number MA 1-21315; Invitrogen, Waltham, MA, United States). Following incubation with primary antibody, the slides were washed 3 × 5 min with PBS and incubated with secondary antibody for 1 h at room temperature (1:1,000, rabbit anti-mouse IgG conjugated with ALEXA 594; Invitrogen, Waltham, MA, United States). After 3 × 5 min washes in PBS, slides were mounted with Vectashield containing DAPI (Vector Laboratories, Burlingame, CA, United States). Epifluorescence images were taken using a Leica DM6000B microscope equipped with a CCD camera and Leica imaging software.

### Native Gel Electrophoresis for Detection of FGF2/IgG and FGF2/Jagged1-Fc Protein Complexes

Native gel electrophoresis was performed using 50 mM MES sodium buffer with 1.3% agarose (ThermoFisher Scientific, Waltham, MA, United States). Adjustments to pH were made, depending on experimental parameters. All gels were run using ice-cold buffer. Purified FGF2, Jagged1-Fc, or IgG were run individually (i.e., free) or as FGF2/IgG or FGF2/Jagged1- Fc complexes to assess their respective mobilities. Human FGF2 was cloned, expressed, and purified in our laboratory (Rao et al., 2015; Miao et al., 2020). Jagged1- Fc was commercial (R&D Systems). Depending on the molar ratios for different complexes, the total protein load for each lane varied between 3.0 and 4.0 µg. After completion of runs, the gels were stained for 30 min at room temperature with Coomassie Brilliant Blue dye and de-stained overnight in 10% acetic acid in deionized water.

### Solid Phase Binding Assays

For solid phase binding assays, polyclonal human IgG was incubated overnight at different concentrations in PBS on ELISA plates (100, 500, or 10,000 ng/ml). Following 3× PBS washes, low molecular weight (18 kDa) human FGF2 was incubated at 4 or 10 ng/ml for 2 h. After additional PBS washes, the amount of bound FGF2 was detected by ELISA (R&D Systems).

### Pulldown of FGF2/Jagged 1-Fc Complexes Using Biotinylated Anti-6x Histidine and Streptavidin-Conjugated Agarose

Growth factor complexes were prepared in 25 µl of PBS (pH 7.4) by mixing 0.5 µg human FGF2 with 7.8 µg of rat Jagged1-Fc-His (catalog # 599-JG, R&D Systems). Notably, these amounts represented an estimated 1:1 molar ratio. As a control, we also incubated separate tubes with 25 µl of PBS (pH 7.4) and 0.5 µg human FGF2 alone (i.e., no Jagged1-Fc). The samples were incubated for 2 h at room temperature (with shaking) and the tubes were then placed at 4°C overnight. The following day, 2 µg of a biotinylated anti-6x His antibody (Invitrogen, Waltham, MA, United States) was added to the complexes and incubated for 2 h with shaking at 4°C. The complexes were then incubated with streptavidin-agarose beads (ThermoFisher Scientific, Waltham, MA, United States) for 48 h, with shaking at 4°C. The beads were pelleted by centrifugation for 10 min at 3,000 × *g*. The supernatant was reserved for ELISA and the pelleted beads were washed 4× with PBS prior to incubation with 0.5% Sodium Deoxycholate (SDC) to disrupt protein-protein interactions. The pulldown supernatant and the detergent soluble fractions were diluted and analyzed by ELISA (human FGF2 Duoset Kit, R&D Systems), according to the manufacturer’s instructions. In some cases, excess heparin sodium (50 µg) was added to the initial 25 µl incubation. To control for possible effects of SDC on ELISA, we added an equivalent amount of SDC to all samples prior to assay.

### Modeling Geometry of Growth Factor/Immunoglobulin Complexes

Docking simulations were made with Patchdock molecular docking server using published crystal structures of IgG, FGF2 and HGF. Separate docking experiments were performed to identify probable associations between: 1) FGF2 and the Fc domain of IgG1, and 2) HGF pan domain and the Fc domain of IgG1. Figures were generated using PyMOL molecular visualization software.

### Statistical Analysis

Statistical analysis was performed with GraphPad Prism software (version 6.0e). Values were expressed as means ± SD unless indicated otherwise. Comparisons of data from individual control and treatment groups were made by unpaired Student’s t*-*test. For experiments comparing multiple treatment groups, we performed One-way ANOVA with *post-hoc* testing. For studies comparing multiple treatment groups across multiple time points, a Two-way ANOVA with repeated measures design was used. Values of *p* ≤ 0.05 were considered statistically significant.

## Data Availability

The original contributions presented in the study are included in the article/supplementary materials, further inquiries can be directed to the corresponding author.
